# Update on Management of Postoperative Atrial Fibrillation After Cardiac Surgery

**DOI:** 10.21470/1678-9741-2019-0164

**Published:** 2020

**Authors:** Rafael de March Ronsoni, Arthur Zanfrilli Marques Souza, Tiago Luiz Luz Leiria, Gustavo Glotz de Lima

**Affiliations:** 1 Universidade da Região de Joinville, SC, Brazil.; 2 Instituto de Cardiologia do Rio Grande do Sul, Porto Alegre, RS, Brazil.

**Keywords:** Cardiac Surgical Procedures, Atrial Fibrillation, Postoperative Period, Prevalence

## Abstract

Postoperative atrial fibrillation (POAF) after cardiac surgery remarkably remains the most prevalent event in perioperative cardiac surgery, having great clinical and economic implications. The purpose of this study is to present recommendations based on international evidence and adapted to our clinical practice for the perioperative management of POAF. This update is based on the latest current literature derived from articles and guidelines regarding atrial fibrillation.

**Table:** 

Abbreviations, acronyms & symbols	
ACCP	= American College of Chest Physicians
AF	= Atrial fibrillation
AV	= Atrioventricular
CABG	= Coronary artery bypass grafting
CCS	= Canadian Cardiovascular Society
POAF	= Postoperative atrial fibrillation

## INTRODUCTION

Atrial ﬁbrillation (AF) remarkably remains the most prevalent event in perioperative cardiac surgery, with an estimated incidence between 20% and 50%, which varies according to electrocardiographic and cardiac monitoring method definition^[1-4]^. Its incidence has continued to grow over recent decades despite the evolution of surgical and anesthetic techniques^[2]^. AF in the postoperative period of cardiac surgery (postoperative atrial fibrillation [POAF]) is associated with worse clinical outcomes, causing a high financial repercussion to health systems^[3-10]^.

Next, we will present recommendations based on international evidence and adapted to our clinical practice for the management of POAF.

### Basic Principles

The primary objectives of POAF management are to maintain hemodynamic stability, control the symptoms, and prevent thromboembolism and recurrence. To achieve these goals, two therapeutic strategies can be used: control of ventricular response and conversion with sinus rhythm maintenance^[11-13]^. As a rule, the best approach is individualized management of each patient correcting complications, such as hypoxia and hydroelectrolytic imbalance (especially magnesium and potassium), which are essential in the prevention and management of POAF. In most cases, the treatment is not different from AF, which occurs in situations not related to surgery. It is important to emphasize the self-limited course of this arrhythmia with up to 30% conversion in two hours. Moreover, 25 to 80% of patients will restore sinus rhythm within 24 hours with or without medications^[14,15]^.

### Rate-Control *vs*. Conversion Strategy in POAF

The advantages of sinus rhythm conversion are decreased cardioversion time, prolonged sinus rhythm maintenance, and reduced hospitalization stay^[16]^. However, some authors suggest ventricular response control, at least initially, because the majority of patients spontaneously revert to sinus rhythm in a short period of time, even without antiarrhythmic medications^[17,18]^. Lee et al.^[16]^ compared medications *vs*. electrical cardioversion for heart rate control in POAF using multivariate analysis in order to control for age, sex, beta-blockers usage, and surgery type. According to the authors, the antiarrhythmic strategy showed a tendency (*P*=0.08) to reduce the time to sinus rhythm reversion. There was also reduced length of hospital stay in the group receiving antiarrhythmic drugs (*P*=0.05). The AF recurrence rate after hospital discharge was similar in both groups (*P*=0.24). The same authors, in a pilot study with a similar design, showed no statistical difference between the two strategies in conversion time from AF to sinus rhythm (*P*=0.8). At the end of the study, 91% of heart rate control arm patients and 96% of control arm rhythm patients were in sinus rhythm, with no statistical significance^[16]^.

The control of ventricular response during AF can be performed with various drugs, including digitalis, beta-blockers, and calcium channel blockers^[2]^. However, the control of response during this period, characterized by increased adrenergic stress, can be particularly difficult^[12,19]^. Beta-blockers are the first-choice medications in postoperative care after cardiac surgery, especially in the presence of ischemic heart disease, unless there are contraindications^[12]^. Importantly, they may be poorly tolerated or contraindicated in the presence of bronchial asthma, decompensated heart failure, and atrioventricular (AV) conduction disorders. Alternatively, non-dihydropyridine calcium channel blockers may be used, except in the presence of AV conduction disorder. Digoxin is less effective because it lacks action on adrenergic tone superimposing the vagotonic effects of the drug on the AV node, but it may be used in the presence of congestive heart failure^[15,20]^.

Given the self-limited course, electrical cardioversion is usually unnecessary when the arrhythmia occurs in the immediate postoperative period. The option of electrical cardioversion for rhythm control should be used in the presence of hemodynamic instability, acute cardiac insufficiency, or myocardial ischemia. It should also be used when the chemical cardioversion fails to revert to sinus rhythm. Anteroposterior paddles and biphasic shock are recommended to optimize the results^[8,21]^. There is no consensus for the best medication to control POAF rhythm. Because of the frequent changes in the hemodynamic pattern of postoperative patients after cardiac surgery, it is difficult to establish a routine treatment. The benefits of pharmacological therapy should be assessed for each patient in relation to adverse effects^[22]^. A randomized double-blind study comparing propafenone with procainamide given intravenously in the postoperative period of cardiac surgery reported that 59% of patients in the propafenone group reverted to sinus rhythm, as opposed to only 18% in the procainamide group (*P*<0.001)^[23]^. In another randomized study, propafenone was compared with amiodarone by Di Biasi et al.^[24]^. Both drugs were equally effective in controlling POAF. However, during the first hour of infusion, the efficacy of propafenone was greater than of amiodarone (44.7% and 19.5% reversion rate, respectively, *P*<0.005). Another study compared propafenone, ibutilide, and a control group in the treatment of POAF. They concluded that only ibutilide decreased the duration of AF (*P*=0.01). Another randomized, double-blind, placebo-controlled trial of ibutilide in 302 patients was performed. Ibutilide (1 mg, intravenously) resulted in a higher rate of atrial arrhythmias reversal when compared to placebo (75% *vs*. 15%, respectively, *P*=0.0001). The efficacy and mean time to conversion were dose-dependent, while conversion rates were higher for atrial flutter than for AF^[17]^. There was a 1.8% incidence of polymorphic ventricular tachycardia in the ibutilide group with a higher incidence in patients with impaired ventricular function^[25]^. Dofetilide, a class III antiarrhythmic agent, showed no difference compared to placebo in the conversion of AF or atrial flutter^[19,26]^. Sotalol has restricted use because it is less effective than other drugs for the treatment of cardioversion. The beta-blocking action is particularly effective for heart rate control in the postoperative period, with a safer profile compared to the drugs previously mentioned^[19,27]^.

The two major complications for using antiarrhythmics in the cardiac surgery postoperative period include ventricular tachycardia (such as *torsades de pointes*) and bradycardia. Risk factors for *torsades de pointes* include electrolyte depletion, bradycardia, and pauses. Excessive diuresis, which often occurs postoperatively, can lead to potassium depletion. Correction of potassium (> 4.0 mEq/L) and magnesium levels before initiation of antiarrhythmic therapy is extremely important. Management of patients with propafenone explicitly recommends a beta-blocker to be given before antiarrhythmic medication for cardioversion of AF in order to prevent rapid AV conduction in the event of atrial flutter^[19,25,28,29]^. One study compared a group of patients who developed POAF in the postoperative period of coronary artery bypass grafting (CABG), receiving amiodarone and early electrical cardioversion, with another control group (digoxin and procainamide or diltiazem). It was concluded that the amiodarone group and early electrical cardioversion were more effective than the control group for the reversion of AF in sinus rhythm patients submitted to elective myocardial revascularization, with safer profile compared to the drugs previously described^[30]^.

In 2009, the first clinical trial reported on the use of vernakalant compared to placebo for the reversal of POAF and atrial flutter. The study included 210 patients from 43 centers in seven different countries. A reversal rate of 47% was achieved in the medication group compared to 14% in the placebo group (*P*<0.001), with a median reversal of 12 minutes. Reversal of atrial flutter was ineffective and only two major side effects were reported (total AV block and hypotension). No ventricular proarrhythmic effects were detected. Therefore, vernakalant may be used in this clinical context, except in patients with severe heart failure, hypotension, and aortic stenosis^[31]^. Considering antiarrhythmic drugs with demonstrated efficacy (amiodarone, flecainide, propafenone, and vernakalant), there was a markedly different response time: vernakalant after 10 minutes, amiodarone after 24 hours, and propafenone and flecainide with intermediate times^[32]^.

Recently, a clinical trial conducted in 23 Canadian and American centers reported on strategies for the management of POAF with clinical stability in 523 patients undergoing CABG and/or valvular surgery. Their results demonstrated that the primary endpoint of hospitalization duration was equal between the two groups (5.1 rhythm control *vs*. 5.0 heart rate control, *P*=0.76). Other interesting data revealed that there was no difference in the presence of sinus rhythm in the control heart rate group (89.9% at hospital discharge and 84.2% at 60 days) and in the rhythm control group (93.5% at hospital discharge and 86.9% at 60 days, *P*=0.14 and *P*=0.41, respectively). There was also no difference in cerebrovascular events, readmissions, and mortality between the two groups. It is worth noting the high crossover rate of both groups, between 20-25%, and a careful analysis of potential changes in results^[33]^.

### Anticoagulation Strategies for the Management POAF

With respect to prevention of thromboembolic phenomena, POAF is associated with a higher risk of stroke, ranging from 1.9% to 18.2%, that emphasizes the use of therapeutic anticoagulation^[21,34,35]^. Our group found a stroke incidence of 11.1% in patients who developed POAF *vs*. 1.9% of incidence in patients who maintained sinus rhythm^[36]^. Patients with left ventricular dysfunction, previous history of thromboembolism, and systemic arterial hypertension are at greater risk for thromboembolic complications. In cases of paroxysmal or persistent POAF for more than 48 hours, anticoagulation should be initiated^[8,12,19,37]^. In addition, in cases of cardioversion, transesophageal echocardiography should be performed to eliminate intracavitary thrombi and then restore sinus rhythm. Anticoagulation is recommended for four weeks, because of the risk of thrombus formation^[38,39]^. The CHA_2_DS_2_-VASc and HAS-BLED risk scores are often used, but validity in postsurgical patients has not been established^[40,41]^. The American College of Chest Physicians (ACCP) recommends the use of anticoagulation particularly for high-risk patients, such as those with a history of stroke or transient ischemic attack that have concomitant POAF. In this clinical scenario, warfarin is the standard treatment. Despite the fact that thrombin and factor Xa inhibitors are indicated for nonvalvular AF, there is little evidence of effectiveness in this clinical context^[38]^. It is important to note that reduction of mortality relative risk with a prescription of coumarin at hospital discharge is 22% at the mean follow-up of six years^[42]^.

In contrast, the use of such therapy increases the risk of bleeding and cardiac tamponade, due to complex coagulation changes, including reduction of coagulation factors, changes in platelet function, and increase in fibrinolytic products related to the procedure^[43]^. However, the merit of anticoagulation in postoperative patients after cardiac surgery should be carefully weighed against the increased risk of bleeding. This risk may even exceed the benefits in reducing stroke in some patients, especially those with the following risk factors: advanced age, uncontrolled hypertension, and previous bleeding history^[44]^.

### Summary of Latest Guidelines

The latest two international guidelines published in 2016 by the European Society of Cardiology and the Canadian Cardiovascular Society (CCS) already address the results of this clinical trial. The summary of guidelines is shown in Tables 1 and 2^[10,33,37]^.

**Table 1:** Therapeutic guidelines for postoperative atrial fibrillation^[37]^.

Class of recommendation I	In the presence of hemodynamic instability, electrical cardioversion is recommended.	Level of evidence C
Class of recommendation IIA	Oral anticoagulation may be considered in patients at risk of stroke, taking into account the risk and benefit in relation to bleeding.	Level of evidence B
Class of recommendation IIA	Antiarrhythmic drugs may be considered for symptomatic patients in an attempt to obtain sinus rhythm.	Level of evidence C

**Table 2:** Prophylactic measures for postoperative atrial fibrillation (POAF)^[10]^.

Strong class of recommendation	POAF may be adequately treated by ventricular response control or cardiac rhythm control	Moderate level of evidence

## CONCLUSION

The latest current literature indicates that a frequency control strategy is as effective as rhythm control in POAF. Therefore, frequency control would be the therapy of choice, confirming data from previous studies that associated POAF with transient pericarditis and autonomic tonus alterations that occur after cardiac surgery and spontaneous resolution over time. Efforts to restore sinus rhythm should be reserved for patients in whom heart rate control cannot be achieved, or for those who are hemodynamically unstable or highly symptomatic.

**Table:** 

Author's roles & responsibilities	
RMR	Substantial contributions to the conception or design of the work; or the acquisition, analysis, or interpretation of data for the work; drafting the work or revising it critically for important intellectual content; final approval of the version to be published
AZMS	Substantial contributions to the conception or design of the work; or the acquisition, analysis, or interpretation of data for the work; drafting the work or revising it critically for important intellectual content; final approval of the version to be published
TLLL	Substantial contributions to the conception or design of the work; or the acquisition, analysis, or interpretation of data for the work; drafting the work or revising it critically for important intellectual content; final approval of the version to be published
GGL	Substantial contributions to the conception or design of the work; or the acquisition, analysis, or interpretation of data for the work; drafting the work or revising it critically for important intellectual content; final approval of the version to be published
